# Carbon Rovings as Strain Sensor in TRC Structures: Effect of Roving Cross-Sectional Shape and Coating Material on the Electrical Response under Bending Stress

**DOI:** 10.3390/s23104601

**Published:** 2023-05-09

**Authors:** Gözdem Dittel, Irem Ecem Cicek, Michael Bredol, Thomas Gries

**Affiliations:** 1 Institut für Textiltechnik of RWTH Aachen University, 52074 Aachen, Germany; 2 Department of Chemical Engineering, FH Münster University of Applied Sciences, 48565 Steinfurt, Germany

**Keywords:** textile reinforced concrete, structural health monitoring, smart concrete structures, sensory carbon rovings, strain sensors

## Abstract

This study investigated the ability of electrically conductive carbon rovings to detect cracks in textile-reinforced concrete (TRC) structures. The key innovation lies in the integration of carbon rovings into the reinforcing textile, which not only contributes to the mechanical properties of the concrete structure but also eliminates the need for an additional sensory system, such as strain gauges, to monitor the structural health. Carbon rovings are integrated into a grid-like textile reinforcement that differs in binding type and dispersion concentration of the styrene butadiene rubber (SBR) coating. Ninety final samples were subjected to a four-point bending test in which the electrical changes of the carbon rovings were measured simultaneously to capture the strain. The mechanical results show that the SBR50-coated TRC samples with circular and elliptical cross-sectional shape achieved, with 1.55 kN, the highest bending tensile strength, which is also captured with a value of 0.65 Ω by the electrical impedance monitoring. The elongation and fracture of the rovings have a significant effect on the impedance mainly due to electrical resistance change. A correlation was found between the impedance change, binding type and coating. This suggests that the elongation and fracture mechanisms are affected by the number of outer and inner filaments, as well as the coating.

## 1. Introduction

Despite their high compressive strength, concrete structures suffer from their low tensile strength. Once the tensile capacity is exceeded and a crack is formed, the structure becomes brittle and the crack propagates in any direction, causing the structure to fail [1,2]. The severity of the structural damage depends on many factors, such as material type, component size, cathodic protection or environmental conditions (e.g., temperature, humidity, soil type and moisture for buried elements). Especially for buried underground systems such as tunnels or concrete pipes, it is difficult to perform continuous maintenance or assess the structural condition, which is very important for the management of transportation, as well as for the gas or fluid distribution systems [3,4].

Bridges are one of the most important infrastructures for goods and personal transport over natural barriers. Their unconstrained service is the key to daily progress and development. However, constant traffic loads and environmental conditions promote crack formation in these structures, presenting a major problem investigated in contemporary concrete research [5]. Currently, manufactured structural concrete parts such as bridge elements or pipes are often steel-reinforced. If the structure is subjected to corrosion, steel reinforcement may lose its tensile strength and its connection to the concrete matrix, causing a fundamental damage in the structure [6]. As an example, chlorides entering the concrete body through damaged joints, seals and transition profiles pose a particular threat to the durability of reinforced concrete bridges. In most cases, corrosion damage of the reinforcement does not become visible until a significant extent of damage has already occurred. The increased traffic volume also causes deficits in some bridges with regard to their lateral force and bending load-bearing capacity in the transverse direction [7,8]. To protect the steel reinforcement against corrosion, these structures require an increased minimum wall thickness, which leads to the overusing of concrete, making the component heavier and increasing transportation costs [1]. Furthermore, an additional monitoring system is usually required to oversee the condition of the structure. However, such systems are often very cost-intensive and can be affected by environmental impacts or structural damage to the concrete [4,9]. This problem may be resolved by integrating the monitoring system into the reinforcement structure.

In recent years, textile-reinforced concrete (TRC) structures have emerged as an alternative to overcome these problems by realizing a single smart composite structure. AR-glass and carbon rovings are the most commonly used reinforcing materials for construction purposes. Unlike conventional steel-bar reinforcement, textile reinforcements can be easily integrated into complex structures; possess high resistance to corrosion, which eliminates the need for high wall thicknesses; and enable us to build thin-walled concrete structures [10,11,12,13,14]. For example, the non-corrosive nature of TRC in bridges enables the reduction of the entire structural weight by approximately 40% through decreased cement use, reduces transport costs for the precast concrete elements and provides a more consistent structure when compared to traditionally reinforced bridges [15,16]. Additionally, its thinner cross-sections allow for a high degree of adaptability to its surroundings and offer the architect and the engineer a wide range of design options [13]. Considering that the cement industry contributes 8% of the global CO_2_ emission, this advantage is of great importance [11,17,18,19,20].

## 2. Carbon Rovings as Strain Sensors

In TRC structures, the bonding mechanism between the concrete matrix and the roving, as well as the stress transfer mechanism between the filaments, plays an important role in the load-carrying capacity of the structure [21,22]. The bundling of the filaments results in the formation of only microscopic gaps between the filaments. Since the concrete matrix cannot penetrate these small gaps, only the outer filaments of each roving have contact with the concrete matrix and thus absorb the load through shear stress of adhesion. However, the absence of tension in the inner filaments limits the load factor to 30–35% [23,24]. As the carried tensile stress increases and the structure begins to crack, some of the outer filaments subjected to the greatest stress also begin to break [12,21]. When a microcrack turns into a macrocrack, in addition to all the outer filaments, some of the inner filaments also break and cause the remaining ones to shift. This structural degradation significantly reduces the load-carrying capacity of the structure and leads to structural damage [12,21,25]. 

The sensory capabilities of carbon rovings can be used to detect this behavior and characterize the structural state of the component [10,21,26,27]. Such a system is based on the continuous measurement of the electrical properties of a carbon roving that correlates with strain. Since a deformation of the construction affects the reinforcement, it causes the rovings to lengthen or shorten, eventually changing their electrical properties. For example, a structure subjected to tensile stress causes the roving to elongate and its cross-sectional area to decrease, resulting in an increase in electrical resistance [27,28,29]. There is a clear difference between the states of strain and damage sensing. The change in electrical resistance observed in strain sensing is mainly reversible, while the change caused by damage is irreversible [21]. Numerous studies on carbon rovings are reported in the literature for their strain-sensing purposes. The main points of the recent studies are summarized in Table 1.

DC-based electrical measurements are commonly used in the literature to detect the mechanical strain or damage in a concrete structure by using carbon rovings as sensors [10,21,23,25,26,28,29,30,31]. Recent investigations recommend AC-based electrical measurements to sense the mechanical strain and damage in the composite structure. The reason is explained by the fact that a single carbon roving, which consists of thousands of filaments, cannot be described as an ideal resistor or an ideal inductor. For a proper characterization of this sensory mechanism, other electrical properties, in addition to the electrical resistance, must be examined, and this is feasible by using AC-based electrical measurements. Uncoated carbon rovings embedded in a concrete matrix have a unique micro-structural mechanism on the basis of abovementioned behavior of outer and inner filaments. A resistor and an inductor serially connected to each other represent a single uncoated carbon roving [32].

The objective of this study was to investigate the mutual effect of different textile binding types and different dispersion concentrations of textile coatings on the strain sensing properties in TRC structures, which differ in their mechanical properties, such as bending tensile strength. Carbon rovings are expected to sense strain, in addition to their reinforcing function. Different treatments of the textile are assumed to affect the electromechanical properties of the carbon rovings. As the optimal trade-off mechanism between the binding type and the coating concentration is being investigated, the goal is to optimize the reinforcement system while maintaining the sensing capabilities.

## 3. Materials and Methods

### 3.1. Textile Manufacturing

Alkali-resistant (AR) glass and carbon rovings were selected for the fabrication of the textile reinforcements. Table 2 lists the properties of the rovings used.

AR-glass rovings, carbon rovings and knitting yarns made of 167 dtex polyester multifilament yarn (PES 167f48) were processed by using the warp-knitting machine BIAXTRONIC of KARL MAYER Technische Textilien GmbH, Chemnitz, Germany. The distance between the roving axes in the textile structure was set to 8.46 mm. Glass rovings were placed both in the weft and warp direction. In the warp direction, some of the adjacent glass roving pairs were replaced with carbon rovings, which were then connected to the glass rovings by the knitting yarns processed in the warp direction. Five different binding types of textiles were used for the examination that were similar to the textiles in a previous work [12] but with a doubled stitching length of 4.76 mm, which is typically used in the commercial warp-knitted textile reinforcements (Table 3).

As shown in Table 3, different binding types of textiles lead to different roving shapes [12,27,28,31]. Pillar and Open Pillar bindings lead to a circular roving cross-section, while Plain and Tricot bindings result in a flat roving cross-section. Counterlaid Tricot, on the other hand, leads to an elliptical cross-section of roving.

While AR-glass rovings are used as the main reinforcement system in both weft and warp directions, carbon rovings form the sensory system in the warp direction, in addition to their contribution to the reinforcement system. This study utilized one carbon roving for the measurements; however, the design of the textile is planned to serve future measurements for water leakage detection in concrete structures by using two adjacent carbon rovings. Based on the configurations using five types of binding and three different percentages of coating, fifteen different test series with six specimens each were obtained. Of the total 90 textile reinforcements, 30 samples were left uncoated.

### 3.2. Textile Coating

The primary purpose of the coating is to increase the mechanical properties of the textile, such as its load-carrying capacity, by bonding the individual filaments within a roving together [36]. Concrete cannot penetrate into the rovings due to its large grain size and is only connected with the outer filaments of a roving in an uncoated reinforcement. When the textile is coated, the inner and outer filaments within a roving are bonded with each other by the coating material, and the load transfer to the inner filaments improves.

Styrene–butadiene rubber (SBR) and epoxy resin (EP) are the most frequently used components in textile coating for construction applications [14]. Compared to SBR coating, the bonding of the filaments provided by the EP coating is better due to its higher penetration capability into the roving, leading to a higher performance [37]. However, the dried EP coating is brittle and fragile, thus preventing the spherical deformation of the textile reinforcement and reducing its plasticity.

In view of such information, water-based SBR was chosen as the coating component. Two different weight/volume coating concentrations, 10% SBR (SBR10) and 50% SBR (SBR50), were selected to show its effect on the strain-detection capabilities of the carbon rovings. To improve the alkali and temperature resistance of the SBR dispersion, 4.75% of curing agent (Lefasol VP 4-5 LF) was added to the coating agent Lefasol VL 90/1 [38]. 

A total of 30 of the textile reinforcement samples were coated with SBR10, while another 30 samples were coated with SBR50. After the coating process, the coating agent was cured for ten minutes at 80 °C; afterwards, it was cured for six minutes at 150 °C in an autoclave. Each textile reinforcement was weighed before the coating and after the curing process. Table 4 shows the final coating amounts on the textile reinforcements, which are determined by the amount of water evaporated in the autoclave. According to the calculations, as expected, the mass fraction of the SBR10-coated textile reinforcements is significantly lower than that of the SBR50-coated textile reinforcements, as shown in Table 4.

### 3.3. Concreting

In total, 90 samples of textile reinforcement were placed into molds of the size 340 mm × 100 mm × 15 mm that were coated with release agent (RECKLI Stripping Wax TL). The standard concrete recipe developed in subproject C1 from the Collaborative Research Centre 532 was used for concreting (Table 5) [39,40].

**Figure 1:**
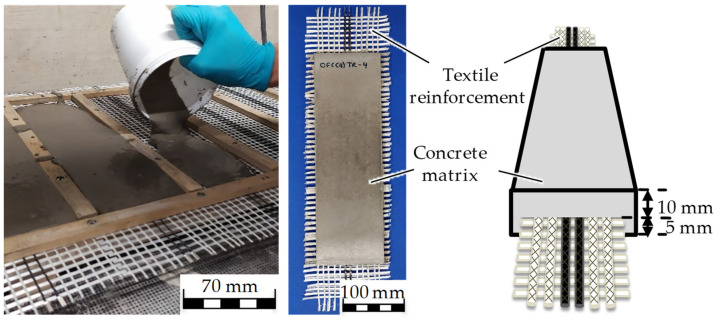
Concreting of textile reinforcements (**left**) and the cured TRC test sample for four-point bending test (**right**).

The solid ingredients, sand, cement, fly ash, silica fume and quartz powder, were mixed, and then the plasticizer and water premix were slowly added to the solid mixture, while mixing continuously. After all the ingredients were homogenized, the final mixture was shaken on a vibration table for two minutes to remove air pockets that formed during the mixing process. The textile reinforcements were concreted, as demonstrated in Figure 1, and vibrated again for five minutes to remove the remaining air pockets and to ensure a full embedment of the reinforcement in the concrete.

After 24 h of curing at room temperature, the samples were then separated from the molds and placed in water at room temperature. The main purpose of this step is to prevent the cracking of the concrete matrix from drying too quickly. After being stored in water for 6 days, the TRC samples were taken out and cured at room temperature for 21 days to complete the hydration process. Lastly, both ends of the carbon rovings were bundled by ferrules to secure the roving shape and to create even connection surfaces for the LCR meter.

The tensile strength of the TRC beam samples was examined in four-point bending tests, while simultaneously measuring the strain sensing capacity of one carbon roving. The textile reinforcement has a distance of 5 mm from the bottom and 10 mm from the top surface of the concrete beam. The aim of this is to position the textile reinforcement in the bending tensile stress zone during the tests and avoid the nominal stress zone.

### 3.4. Experimental Setup

**Figure 2:**
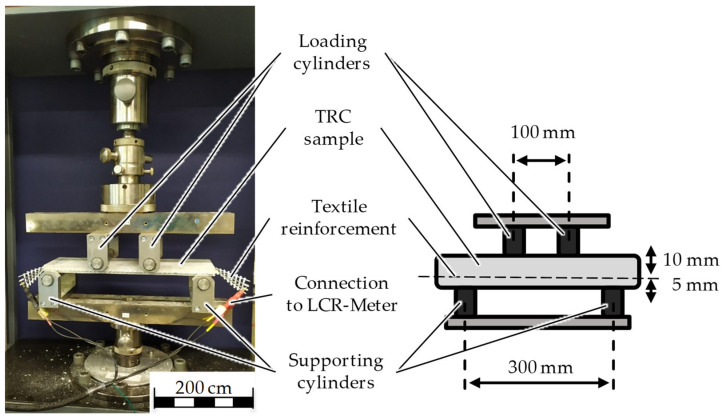
Experimental setup of electrical measurements during the four-point bending test.

The setup is designed to continuously measure the electrical properties of the carbon rovings during a four-point bending test to investigate and compare their strain-sensing capacity. The four-point bending tests were performed according to the DIN EN 1170-5 standard on the universal testing machine Z100 of ZwickRoell GmbH & Co. KG, Ulm, Germany, as shown in Figure 2.

The loading cylinders have a distance of 100 mm from each other and remain fixed during the test. The supporting cylinders, on the other hand, have a distance of 300 mm from each other and can move upwards according to the test settings. The test speed is set to 1.8 mm per minute. The TRC sample is oriented and mounted in the apparatus in such a way that the distance between reinforcement and supporting cylinders is 5 mm. Both ends of one carbon roving of the textile reinforcement are connected to the LCR-Meter Wayne Kerr 43,200 to observe the change in the electrical impedance.

**Figure 3:**
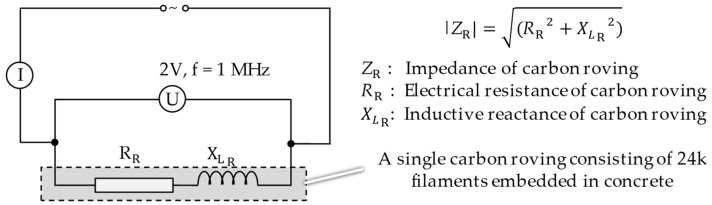
Electrical circuit of a carbon roving to measure the change in electrical impedance [42].

The bending tests and the electrical measurements were conducted at a standard climate, in accordance with DIN EN ISO 139 [41], and started simultaneously when a preliminary force of 5 N was reached. The establishment of a preliminary force allows us to eliminate the differences in sample thicknesses. An RMS voltage of 2 V and a frequency of 1 MHz were applied to one carbon roving from the LCR-Meter (Figure 3).

The change in the electrical impedance, which is affected by the electrical resistance (*R*), inductance (*L*) and capacitance (*C*), was measured continuously as the length of the filaments within a roving and the filament diameter changed with the strain. Once the force on the TRC after passing its maximum dropped again to 75% of the maximal force, the four-point bending test and the electrical measurement were stopped simultaneously. The basic measurement accuracy of the used LCR-Meter was 5% at the applied frequency. The measurement range for the impedance and resistance was between 10 µΩ and 100 GΩ; for the inductance, it was between 1 nH and 2 kH; and for the capacitance, it was between 0.01 pF and 1 F [43].

## 4. Results

The test results obtained from the mechanical investigation consist of two parts: mechanical results of the four-point bending test and electrical results of the impedance measurement, which are presented in Section 4.1 and Section 4.2, respectively. While the mechanical results of the four-point bending test focus on the bending tensile strength and elongation of the TRC samples, the electrical results consist of a visualization of the changes in electrical impedance of the carbon rovings.

### 4.1. Mechanical Results under Strain

During the four-point bending test, cracks occurred in the mechanically loaded TRC samples. In addition to this intended crack formation, it was observed that the concrete matrix separated from all the SBR50-coated rovings with a flat cross-sectional shape (Plain and Tricot binding) at the end of the bending test. Since this separation was unexpected, these TRC samples were considered for further investigation to analyze the effect of the failure mode. Potential root causes for the results are discussed in Section 5.

**Figure 4:**
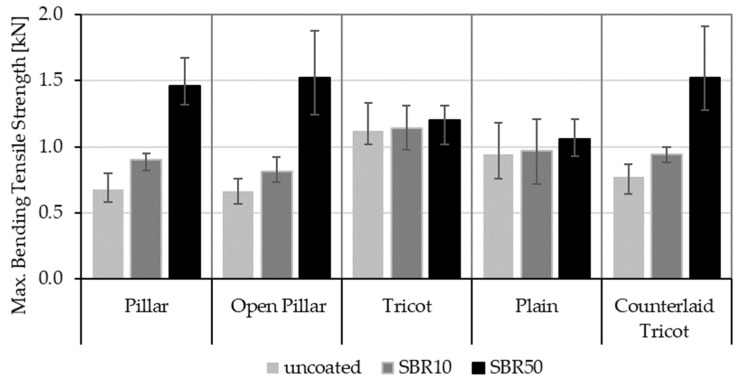
Maximum bending tensile strength of TRC samples under four-point bending test.

The flexural tensile strength of each TRC sample was measured during the four-point bending test. Figure 4 compares the average values for maximal bending tensile strength of the TRC samples.

As also demonstrated in earlier studies, the maximum load-carrying capacity of TRC samples varies depending both on the coating concentration of the textile reinforcement and the binding type of the textiles [12,44]. In this study, all binding types were created with a doubled stitching length of 4.76 mm, unlike in the previous works. The change in this parameter affects the results slightly in comparison to those of the previous tests.

In the case of uncoated textile reinforcement, the rovings with a flat cross-sectional shape (Tricot and Plain binding) lead to a higher bending tensile strength than other ones. This is followed by the elliptical cross-sectional shape of the Counterlaid Tricot. Although it does not appear to have a strength as high as the flat cross-sectional shape, it performs better than the circular cross-sectional shapes of the Pillar and Open Pillar.

However, this situation changes when the SBR coating is applied to the textile reinforcement. A consequential increase in load-carrying capacities is observed for all binding types, but the growth is much higher in the circular and elliptical cross-sectional shapes of roving. For the SBR10-coated TRC samples, the Pillar binding shows the greatest improvement in tensile strength, with a value of 1.32 times higher than in its uncoated state. With the application of the SBR50 coating, the Open Pillar shows the highest increase, with a value of 2.3 times the base value. Meanwhile, the lowest increase in both categories is Tricot, with ratios of 1.02 and 1.07 times, respectively.

**Figure 5:**
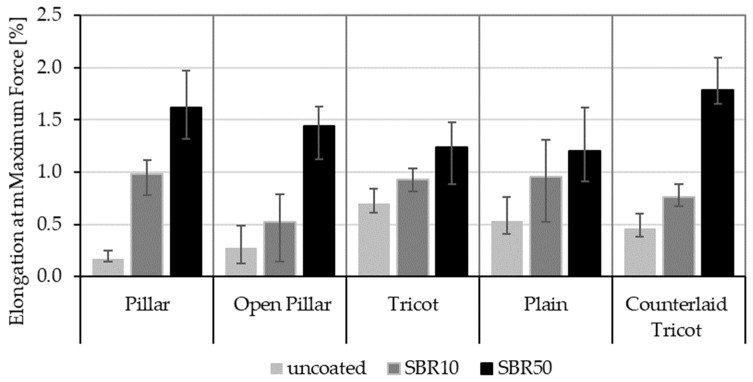
Elongation of TRC samples at maximum force.

Moreover, the maximum elongation percentages of the TRC samples are measured under the applied force to compare the ductility of the samples. Figure 5 shows the elongation of all TRC samples at maximum force.

In all cases, the increased coating concentration improved the ductility of the TRC samples and, thus, the maximum elongation. As with the load-carrying capacities of the samples, the increase in elongation is larger in rovings with circular and elliptical cross-sectional shapes. These results also form the basis of the electrical investigations presented in the next section, as the elongation of the rovings directly affect the electrical impedance of the carbon rovings.

In summary, as also proven in previous studies [12,31,44], the mechanical results under strain indicate that rovings with a flat cross-sectional shape show greater flexural strength when uncoated, while rovings with a circular or elliptical cross-sectional shape become more effective when coated. Thus, SBR50-coated TRC samples (Pillar, Open Pillar and Counterlaid Tricot bindings) show the best mechanism for the mechanical purposes at this stage.

### 4.2. Electrical Results under Strain

Bending of the samples by the applied force causes the filaments within a roving to elongate and, eventually, the filament diameter to decrease due to the lateral contraction. This structural change affects the electrical properties of the conductive carbon rovings [27,29,32]. To compare the effects of the binding types on the average impedance change, Table 6 divides the obtained results into three main categories: uncoated, SBR10-coated and SBR50-coated TRC samples. Each diagram represents the results obtained from one single sample of a test series. It must be noted that the curve slope and the amount of electrical resistance changes contribute more than 99% to the observed impedance changes, since all changes in measured inductance are at the µH level, and all changes in measured capacitance are at the nF level; thus, they only marginally affect the impedance.

The mean value of the impedance change and the corresponding standard deviation are given in Table 7 to include the measurement results of all samples of a test series.

As shown in Table 6, all binding types are able to capture strain changes regardless of coating concentration. As expected, the initial impedance values of the uncoated textiles are lower than those of the coated textiles. Different binding types do not affect the initial values for the uncoated samples significantly. For the coated samples, these values deviate depending on the binding type. The standard deviation within a series is highest for the SBR50-coated samples. The curve slopes within a series show comparable trends.

The largest impedance change is measured with the binding types Pillar and Counterlaid Tricot for the uncoated TRC samples, while Pillar shows the largest change for SBR10 and Counterlaid Tricot for the SBR50-coated samples. The binding types that exhibit the largest change in impedance in coated states are identical to the ones that show the highest changes in mechanical properties.

SBR50-coated TRC samples show the highest change in impedance. This observation is irrespective of the binding type. However, SBR10 coating correlates negatively with the impedance change compared to uncoated samples.

Carbon rovings successfully detect the strain on the TRC structure. SBR50-coated TRC samples show the largest change in impedance and, thus, the highest sensitivity to strain. Otherwise, changes in impedance do not reflect the relation of mechanical changes in uncoated and SBR10-coated samples.

## 5. Discussion

### 5.1. Mechanical Investigations under Strain

The results of the four-point bending test show that the bending tensile strength of the TRC samples changes with both the cross-sectional shape of the rovings and the coating concentration of the textiles.

When uncoated, the rovings with a flat cross-sectional shape show the highest tensile strength. This was previously explained in Refs. [12,27] by the different adhesion forces between the concrete matrix and the textile reinforcement, which highly depend on the surface area of the rovings. In the uncoated state, the load is transmitted through the adhesion forces between the outer filaments of the rovings and the concrete matrix. While the outer filaments are forced to carry this load, inner filaments remain stress-free. In the case of rovings with a flat cross-sectional shape, the number of outer filaments connected in the concrete matrix is higher than that of the rovings with a circular or elliptical cross-sectional shape. Thus, rovings with a flat cross-sectional shape possess a more stable bond to the concrete matrix. The increase in contact area leads to a higher adhesion force and, thus, a better tensile strength [12,27,44].

When SBR coating is applied to the textile reinforcement, the tensile strength of the TRC sample increases gradually with the increasing concentration of coating. The coating ingresses through the micro-gaps of the filaments and forms a bond between the inner and outer filaments. This enables a better stress distribution along all fibers and, thus, a gradual increase in the bending tensile strength. In addition, it is noticeable that rovings with circular and elliptical cross-sectional shapes exhibit a significantly larger increase in tensile strength when compared to the rovings with a flat cross-sectional shape. This larger increase is attributed to the higher number of non-active inner filaments within the rovings that become active when coating material connects these to the outer filaments. These rovings demonstrate a higher number of active inner filaments than rovings with a flat cross-sectional shape and thus have a larger increase in tensile strength [12,44].

**Figure 6:**
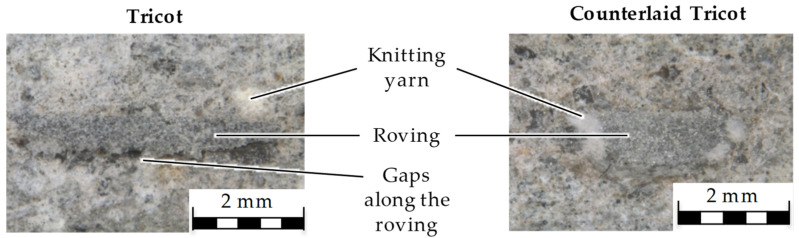
Microscopic investigation of SBR50-coated flat roving (**left**) and elliptical roving (**right**).

It is noticeable that after reaching the maximum tensile strength, the drop in flexural strength takes place without any elongation. After the microscopic investigations, the sudden decrease in the flexural strength is assumed to be due to the accumulated air pocket formation on the roving surface as shown in Figure 6.

Such gaps between the textile reinforcement and the matrix may directly affect the bonding forces and lead to a loss in strength. The reason for the air-pocket formation is assumed to be the small grid openings due to the flat cross-sectional shape of rovings and knitting yarns between the rovings, which prevent the easy penetration of the concrete matrix through the grid opening.

Additionally, the relations between the Tricot and Plain binding results were reversed compared to the previous studies. The reason can be outlined by the larger stitching length of 4.76 mm, which arranges wider rovings with a flatter cross-sectional shape in all textile types, resulting in smaller grid openings. Since the distance between the roving axes is 8.46 mm for all textile types investigated in this study, the textile with Plain binding has a relatively close grid opening (see Table 3), which may result in a sieve effect for the concrete. The weak bond between the textile reinforcement and the concrete matrix caused a separation of the concrete from the reinforcement with Tricot and Plain bindings towards the end of the bending test.

### 5.2. Electrical Investigations under Strain

The bending of the TRC samples causes the rovings to elongate and break. Considering the elongation of the TRC samples, it is expected that the measured change in impedance increases with the increasing concentration of coating. However, this relationship is only shown between the different concentrations of coating. The comparison of the impedance changes between the uncoated and the SBR10-coated TRC samples reveal that it is not yet possible to establish this correlation between the coating concentration, resulting in increased elongation, and the change in impedance. This situation demonstrates that not only the elongation plays an important role in resistance change but also the fracture of the filaments. At this point, it is assumed that a clear relationship cannot be established between the impedance change and coating concentrations of the carbon rovings, since it is not yet possible to distinguish which of these two factors, elongation or fracture, cause the greater change in resistance and thus in impedance.

Regardless of the total change in resistance, a situational analysis is conducted by plotting the curve of the changes in impedance measured from one representative carbon roving from each set on the load–deformation curve of the structure. This may reveal a sample-specific correlation for the coating by considering the factors elongation and fracture.

In the case of the uncoated reinforcement, the changes in electrical impedance reflect a correlation between the shape of the roving cross-section and the elongation of the rovings. When the number of outer filaments is low, as in the Pillar or Open Pillar binding types, the impedance increases visibly in the earlier stages of the macro-structural response of the TRC beams under flexural load. In the case of the uncoated samples with a circular cross-section, the impedance—in particular, the electrical resistance—of the carbon roving increases during the bending test. Since there is no stress distribution towards the inner filaments of uncoated rovings, this increase in resistance is related only to the elongation of the overall length of the outer filaments. Apart from the steady increase in resistance, the change is more distinct and rapid at certain points, corresponding to the formation of major cracks on the samples. This indicates the fracture of the outer filaments due to the high stress. Fracture of the rovings causes a reduction in the cross-sectional area of the roving, leading to a very rapid increase in resistance and, thus, in impedance. The measurement of these two parameters demonstrates that crack formation of the structure can be detected precisely.

When the number of outer filaments is high, as in Tricot and Plain binding types, the increase in impedance remains small until the cracks have expanded considerably and then rises sharply. It is assumed that the break of the outer filaments decreases, and the elongation of the inner filaments increases the electrical resistance with a closed potency, and, thus, an almost straight curve slope is observed in the beginning of the bending tests. After all outer filaments are broken, the remaining inner filaments pull out until break, resulting in an abrupt increase in the electrical resistance/impedance. In the case of the Counterlaid Tricot binding, the pull-out mechanism of the inner filaments starts slightly earlier since the number of outer filaments is lower, and, thus, all outer filaments break earlier.

The coating causes more filaments to elongate and break together at the same time, resulting in a relatively unsteady increase in resistance. The coating distributes the mechanical stress more evenly along the roving so that the damage does not occur on one cracking point but rather distributes itself into multiple smaller cracks inside the concrete element. However, a clear fracture point, as in the uncoated state, is not observed. On the other hand, the impedance of the SBR10-coated TRC samples increases constantly, while the impedance of the samples coated with SBR50 often decreases after a certain point. It is assumed that a reason for this situation could be the shortening of the filaments at the cracked zones. Table 8 shows the total crack widths of TRC samples after the four-point bending test.

The crack widths are larger in the SBR50-coated TRC samples. The wide crack formation in the concrete matrix, which normally holds the elongated filaments together during the four-point bending test, may have caused the carbon filaments to shrink back. This correlates with maximal elongation. If a structure can bend more before breaking, the energy release is bigger, resulting in wider cracks.

## 6. Conclusions

The investigations performed on the TRC samples show that the binding type and the concentration of coating applied to the textile reinforcement have a significant effect on the mechanical properties of the structure.

During the mechanical tests in the uncoated and SBR10-coated cases, the rovings with a flat cross-sectional shape showed a very high tensile strength and a high elongation compared to the other binding types. However, the rovings with circular and elliptical cross-sectional shapes revealed better results in tensile strength and elongation when coated with SBR50.

The separation of the concrete matrix from the SBR50-coated rovings with flat cross-sectional shapes and the not prominent increase in tensile strength are explained by microscopic examinations.

The optical inspection showed that air pockets are formed in the concrete matrix. Such a formation promotes the reinforcement to detach from the concrete matrix with the increasing load by compromising the ability to transfer stress. The potential origin of the air pockets could be the concrete matrix that could not penetrate through the small meshes of the textile during sample preparation. To prevent this, further investigations should address this issue by increasing the vibration time during concreting or even consider another concrete formulation such as self-compacting concrete.

Electrical measurements during the four-point bending test give a direct estimation about the condition of the structure in all cases. Even though the increase in impedance is low, an estimation of the structural strain on the textile is possible. The data show that the total impedance change is higher for the TRC samples which are coated with SBR50 than for the others. However, the effect of the binding type on the sensory properties is not yet fully understood.

According to these results, textile reinforcements with circular or elliptical cross-sectional shapes and a higher coating concentration are recommended for further research, since the coating of the textile reinforcement increases the flexural strength of the TRC sample and also the strain-sensing capabilities of the carbon rovings.

## Figures and Tables

**Table 1:** The literature research on carbon rovings as strain sensors.

Aim	Binding Type	Coating	Sample Configuration	Electrical Configuration	Insights/Observations	Ref.
Investigation of self-sensing potential under repeated (cyclic) loading	Pillar	uncoated	AR-glass/carbon-based TRC beam	Wheatstone bridge (DC)	Structure senses the mechanical load but loses linearity over time at the macroscopic level.	[26]
Investigation of self-sensing potential under monotonic loading	Pillar	uncoated	AR-glass/carbon-based TRC beam	Wheatstone bridge (DC)	Structure is able to sense the structural behavior at the macroscopic level.	[29]
Investigation of sensitivity of electrical resistance	Tricot Pillar	uncoated	Single C-roving, C-roving in concrete, and C-roving in polymer composite	DC Circuit	Resistance shows good correlation with strain. Binding type contributes to flexural behavior and electrical response.	[30]
Searching a correlation between electrical changes with micro- and macrostructural effects	Pillar	uncoated	AR-glass/carbon-based TRC beam	Wheatstone bridge (DC)	Changes in structural behavior under monotonic flexural loading process can be characterized over the loading range by the changes in the electrical reading of the carbon rovings.	[10]
Investigation of the relationship between the electrical response and the structural behavior to sense damage under cyclic mechanical load	Pillar	uncoated	AR-glass/carbon-based TRC beam	Wheatstone bridge (DC)	Sensory textile can differ between damage and internal microstructural effects.	[21]
Investigation of elasticity of carbon rovings embedded in concrete	Pillar	uncoated	Biaxial warp-knitted fabric with carbon and AR-glass	Wheatstone bridge (DC)	Change of resistance and elasticity is similar to metal. Tensions of C-rovings are measurable through elasticity, not following Hook’s law if higher than 0.2%.	[28]
Characterization of material properties of textile reinforcement equipped with carbon rovings	Tricot Plain Pillar	uncoated	Biaxial warp-knitted fabric with carbon and AR-glass	Wheatstone bridge (DC)	Binding types showed high tensile and shear forces. Four-point bending test demonstrate that k-factor aligns with yarn, plastic composite and concrete matrix.	[31]
Investigation of the effects of coating on the self-sensing capabilities under cyclic mechanical loading	Pillar	SBR15	AR-glass/carbon-based TRC beam	Wheatstone bridge (DC)	Coating enhances mechanical performance but restricts the ability to sense the structural state and identify severity of the cracks.	[23]
Characterization of the electrical properties of carbon rovings due to mechanical strain and cracking at various structural states	Pillar	uncoated	AR-glass/carbon-based TRC beam	AC circuit	A carbon roving in a TRC beam is characterized by an RL electrical circuit, and the electrical properties (R and L) are affected by the geometrical properties of the roving and by the concrete matrix.	[32]

**Table 2:** Properties of AR-glass and carbon rovings [33,34].

**Properties**	**AR-Glass Rovings**	**Carbon Rovings**
Manufacturer	Owens Corning	SGL Group
Number of filaments (k)	3.2	24
Filament diameter (µm)	19	7
Density (g/cm^3^)	2.68	1.81
Fineness (tex)	2400	1600
Tensile strength (GPa)	1.7	5
Young’s modulus (GPa)	72	270

**Table 3:** Binding types and different geometries of textile samples. Drawings based on [35].

**Binding Type**	**Textile Structure**	**Knitting Sketch**	**Roving Shape**
Pillar	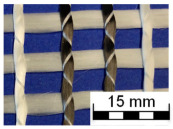	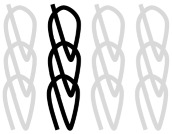	Circular 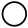
Open Pillar	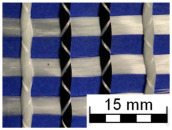	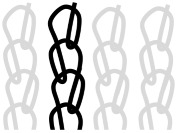	Circular 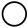
Tricot	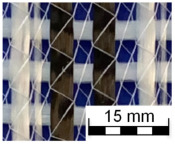	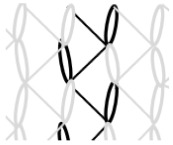	Flat 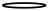
Plain	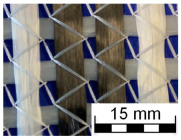	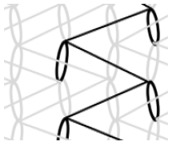	Flat 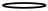
Counterlaid Tricot	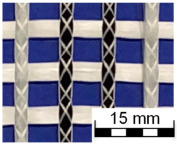	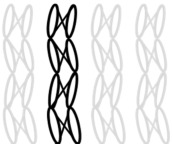	Elliptical 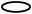

**Table 4:** Final coating amount on the SBR10- and SBR50-coated textile reinforcements.

Coating Type	Textile Coating Percentages by Weight (wt%)				
	Pillar	Open Pillar	Tricot	Plain	Counterlaid Tricot
SBR10	3.1	2.7	3.6	2.7	3.5
SBR50	14.6	11.0	18.4	20.5	19.4

**Table 5:** Proportions of the components used for concrete mixture [39,40].

	Cement CEM I 42.5 R	Sand 0.2–0.6	Water	Fly Ash	Silica Fume	Quartz Powder	Plasticizer	Total
Quantity (kg/m^3^)	490	713	280	175	35	500	7	2200

**Table 6:** Comparison of electrical impedance changes of different binding types and coating concentrations (load and impedance change versus displacement).

	Uncoated	SBR10	SBR50
**Pillar**	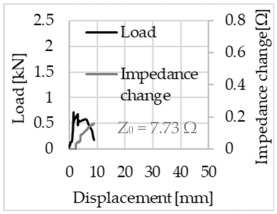	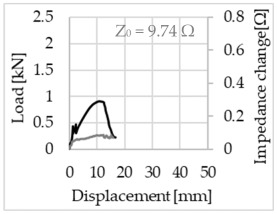	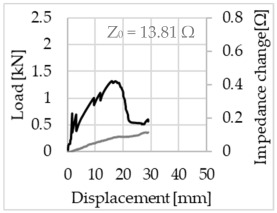
**Open Pillar**	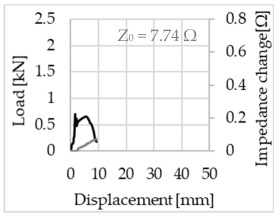	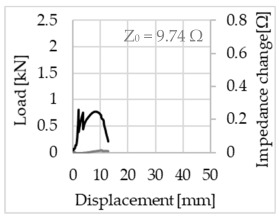	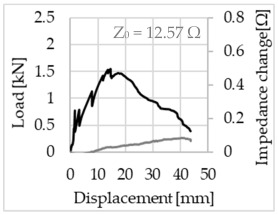
**Tricot**	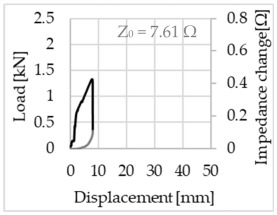	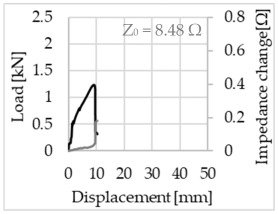	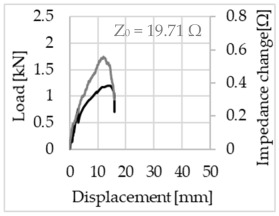
**Plain**	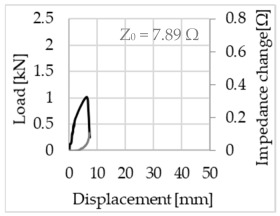	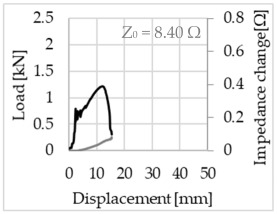	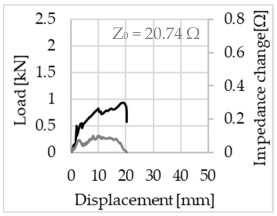
**Counterlaid Tricot**	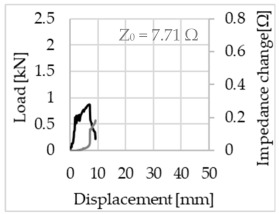	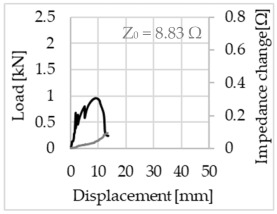	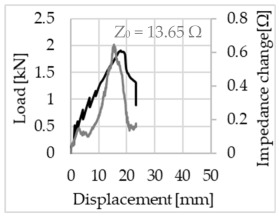

**Table 7:** The mean value of the impedance change and the related standard deviation for 90 samples.

Binding Type	Mean Value of Impedance Change ± Standard Deviation		
	Uncoated	SBR10	SBR50
Pillar	(0.22 ± 0.14) Ω	(0.13 ± 0.08) Ω	(0.24 ± 0.35) Ω
Open Pillar	(0.11 ± 0.08) Ω	(0.09 ± 0.17) Ω	(0.50 ± 0.68) Ω
Tricot	(0.13 ± 0.04) Ω	(0.12 ± 0.06) Ω	(0.28 ± 0.24) Ω
Plain	(0.12 ± 0.03) Ω	(0.06 ± 0.02) Ω	(0.37 ± 0.37) Ω
Counterlaid Tricot	(0.22 ± 0.10) Ω	(0.08 ± 0.04) Ω	(0.56 ± 0.89) Ω

**Table 8:** Total crack widths of TRC samples after the four-point bending test.

Binding Type	Total Crack Width (cm)		
	Uncoated	SBR10	SBR50
Pillar	0.108	0.162	0.285
Open Pillar	0.110	0.118	0.504
Tricot	0.052	0.092	-
Plain	0.037	0.192	-
Counterlaid Tricot	0.087	0.128	0.229

## Data Availability

Not applicable.
